# Percentage of Discharged COPD Patients with Exclusion Criteria for Participation in Outpatient Pulmonary Rehabilitation †

**DOI:** 10.3390/jcm14092863

**Published:** 2025-04-22

**Authors:** Hnin H. Oo, Osama Elsankary, Diahann K. Wilcox, Antarpreet Kaur, Jane Z. Reardon, Jose A. Soriano, Debapriya Datta, Richard ZuWallack

**Affiliations:** 1 Department of Pulmonary, Critical Care and Sleep Medicine, University of Connecticut, Farmington, CT 06030, USA; 2 Frank H. Netter School of Medicine, Quinnipiac University, North Haven, CT 06473, USA; 3 Department of Pulmonary, Critical Care and Sleep Medicine, St. Francis Hospital, Hartford, CT 06105, USA

**Keywords:** chronic obstructive pulmonary disease, pulmonary rehabilitation, eligibility, comorbidity

## Abstract

**Background/Objectives**: Despite documented benefits across multiple outcome areas, referral and uptake into pulmonary rehabilitation (PR) following discharge after an exacerbation of chronic obstructive pulmonary disease (COPD) is low in many health care systems. Surveys documenting this underutilization may ignore the fact of disease severity or comorbidity severe enough to make many patients ineligible based on accepted selection criteria for the intervention. The aim of this study was to evaluate the magnitude of non-eligibility for PR following discharge after a COPD exacerbation. **Methods**: Medical records of COPD patients discharged over a one-year period in two hospitals were reviewed. Records from 353 patients discharged home were reviewed by six clinicians with experience in respiratory medicine and/or PR, three at each hospital. **Results**: The mean age of the total sample was 71 ± 12 years; 53% were female. Full concordance (all three reviewers agreed on the eligibility or non-eligibility of each patient) was 73%. Our eligibility criterion (two of three reviewers agreed) for PR was 39%. Categories (%) of non-eligibility criteria included the severity of medical condition(s) (44%), cognitive problems, psychiatric disease or substance abuse (24%), incorrect diagnosis (18%), institutionalized post-discharge (9%), and language barriers (4%) (patients may have been placed into more than one criteria category). **Conclusions**: Our study indicates that a majority of patients with clinical diagnoses of COPD discharged following exacerbations may not be appropriate referrals to PR based on accepted inclusion and/or exclusion criteria for the intervention. However, even after taking this into account, PR uptake is still critically underutilized.

## 1. Introduction

Pulmonary rehabilitation (PR) typically results in substantial improvements in dyspnea, exercise tolerance, and health status in patients with COPD [1]. Additionally, when PR is initiated after a hospital discharge for COPD, it may reduce mortality and subsequent hospitalization risk [2]. Recognition of these benefits led to the inclusion of PR in national and international clinical guidelines for COPD and coverage by many insurance carriers, including Medicare. However, low levels of referral, uptake, and completion remain problematic across many health care systems [3,4], especially following discharge after COPD exacerbations—when patients are most vulnerable for further adverse outcomes. For example, Spitzer and colleagues determined that of 223,832 Medicare patients hospitalized with principal diagnoses of COPD or acute respiratory failure, only 732 (0.3%), 3321 (1.5%), and 6111 (2.7%) initiated PR at 1, 3, and 12 months, respectively [5].

Several reasons have been suggested to explain PR underutilization, including travel and transport barriers [6,7], inadequate referral [7], lack of perceived benefit from the intervention [6,7], lack of knowledge of PR [7], current cigarette smoking [6], wait times or post-discharge timing [6,8], anxiety over starting PR [2], and inadequate integration between hospital and PR services [9]. Recognition of this underutilization and of potential barriers to PR has led to policy recommendations by the American Thoracic Society (ATS) and European Respiratory Society (ERS) directed towards increasing its utilization in individuals with chronic respiratory diseases [10].

While audits and published reports correctly point out the low uptake of PR in the post-discharge setting, some COPD patients may not have been eligible for PR in the first place, based on inclusion and/or exclusion criteria for participation. Removing from the denominator those patients who did not meet PR selection criteria would increase uptake percentages and provide a more accurate estimate of the problem. Accordingly, we retrospectively determined PR eligibility using standard inclusion and exclusion criteria in all COPD patients discharged over a one-year period in two hospitals: one in an urban location and another in a suburban location in Connecticut. The purpose of this study was to evaluate the magnitude of non-eligibility for PR following discharge after a COPD exacerbation.

## 2. Methods

After Institutional Review Board approvals, we reviewed electronic records of patients with discharge diagnoses of COPD, COPD exacerbation, or respiratory failure associated with a COPD exacerbation (J43.0, J43.1, J43.2, J43.8, J43.9, J44.0, J44.1, and J44.9). A review was conducted on those discharged from the hospital over a one-year period beginning on 6 January 2022 in two acute care, teaching hospitals: one in an urban location (Hospital A) and the other in a suburban location (Hospital B) in Connecticut. If more than one admission in the above time frame was present, only the first was reviewed. Six physician or nurse practitioner clinician reviewers with extensive experience in COPD and/or pulmonary rehabilitation performed reviews in these two hospitals, three at each site.

Eligibility for PR was based on the patient meeting inclusion and not meeting exclusion criteria for participation, based on recommendations published in an American Thoracic Society/European Respiratory Society statement on PR [1]. Inclusion criteria were persistent respiratory symptoms such as dyspnea or fatigue and/or functional status limitations despite otherwise optimal therapy. Exclusion criteria were a condition that would either (a) place the patient at substantially increased risk during pulmonary rehabilitation or (b) substantially interfere with the PR process. Medical conditions that might preclude eligibility, provided the patient met either condition (a) or (b) above, could be primary respiratory disease, a comorbid condition, extreme frailty, psychiatric disease, cognitive problems, or substance abuse. Direct discharge from the acute care hospital to another facility, such as to a skilled nursing facility or to incarceration, was considered a criterion for non-eligibility. Death within one month of hospital discharge was considered an exclusion criterion under the disease severity category. As the PR centers within driving distance of the two hospitals only had English-speaking staff, if a language barrier was present and sufficiently interfered with the PR process, this was considered an exclusion criterion.

Of note, as information recorded in the medical record at the time of hospitalization usually did not include specific information on symptoms or functional status severity, our analysis assumed patients severe enough to be hospitalized met one or both criteria. As chronic respiratory disease is a required inclusion criterion, we considered a mistaken diagnosis of COPD (from record review) as an exclusion criterion. Additionally, we did not consider the presence or absence of documented airflow limitation (forced expiratory volume in one second divided by forced vital capacity (FEV1/FVC) < 0.70) in our determination of eligibility, as these results were inconsistently present in electronic records.

The record review focused on the following information:

1. Patient demographics: age, sex, race, socioeconomic status (our marker was Medicaid insurance or no insurance)
2. Was the discharge diagnosis (i.e., COPD) correct, based on data recorded at the time of hospitalization?
3. Were either of the above exclusion criteria (anticipated substantial risk by participating or a condition that would substantially interfere with the PR process) present?
4. If exclusion criteria were present, they were categorized into the following groups:
  - Illnesses, debility, or frailty either too great for participation or would put the individual at excessive risk (death within one month of discharge was placed in this category, while those who died (n = 3) while in hospital were not included in the analysis);
  - Cognitive, psychiatric, or substance abuse issues that would preclude full PR participation;
  - Institutionalized post-discharge;
  - Language or communication barrier sufficient to interfere with rehabilitation or put the patient at undue risk.

Our surrogate marker for low socio-economic status (SES) was Medicaid or Medicare–Medicaid insurance or no insurance. Race was determined by self-identification in hospital records. Ethnicity was not evaluated.

Eligibility or non-eligibility was independently determined by three clinicians at each hospital, based on review of available medical record entries corresponding to the time of the index hospitalization. Each hospital review was conducted by two physicians and one nurse practitioner. Full concordance among the three reviewers at each hospital was considered present if all three reviewers at the site agreed (i.e., either YES-YES-YES or NO-NO-NO). However, our eligibility or non-eligibility decision was based on agreement by at least two of the three reviewers. Three additional determinations of eligibility were also made, as follows: (1) after eliminating from the analysis those with an incorrect diagnosis of COPD; (2) after eliminating only those with an incorrect diagnosis as the *sole* criterion for non-eligibility; and (3) after elimination of a language barrier as an exclusion criterion.

SAS version 9.4 (Cary, NC, USA) was used for descriptive analyses and for comparisons between hospitals.

## 3. Results

Records from 217 discharges following hospitalizations were reviewed in Hospital A and 136 in Hospital B, giving a total of 353 reviews. Patient characteristics for each of these two hospitals and the total group are given in Table 1. Age and sex were not significantly different in the two hospitals. However, Hospital A (the urban hospital) had a higher percentage of patients with low SES and a higher percentage of patients self-identified as Black (*p* < 0.0001 and *p* = 0.0001, respectively).

Full concordance (i.e., all three reviewers at each site were in agreement, either YYY or NNN) was present in 73% of the 353 reviews: 69% in Hospital A and 79% in Hospital B (*p* = 0.03). Kappa statistics to assess the level of agreement between rater pairs at each hospital ranged from 0.46 to 0.75.

Eligibility for PR (i.e., two of the three reviewers agreed the patient would be a candidate) was 39% for the entire group; 38% for Hospital A and 40% for Hospital B. These percentages were not significantly different (*p* = 0.72). With respect to those determined to be non-eligible for PR (i.e., 61%), full concordance among the three investigators (i.e., NNN) for the entire sample was 174 of 217 ineligible (80%); for Hospital A and B these percentages were 68% and 84%, respectively.

Categories of non-eligibility for the total group and each of the two hospitals are given in Table 2. The two hospitals differed significantly in this categorization, with Hospital A having fewer incorrect diagnoses of COPD and a higher percentage of institutionalization post-discharge (*p* < 0.0001 and *p* = 0.0007, respectively).

Of the total group, 64 (18%) had an incorrect COPD diagnosis as at least one non-eligibility criterion and 27 (8%) had this as the sole criterion. After eliminating these 64 patients from the analysis, PR eligibility in the total group was 45%, and after eliminating just those 27 who had this as the sole criterion of PR eligibility, it was 42%. Five of the fourteen patients with a language barrier as an exclusion criterion had this as the sole exclusion criterion. After considering these five patients eligible, eligibility for the total group remained at 39%.

## 4. Discussion

The process of pulmonary rehabilitation involves several steps: an initial assessment by a referring clinician; referral to PR (although some patients may self-refer); attendance at the center by the patient; a decision by the PR staff that that patient is indeed a reasonable candidate for PR; and meaningful participation by the patient in PR. Barriers can occur in any of these steps. Our study focused only on the first step—whether a patient at the time of discharge after a COPD exacerbation would be a potential candidate for PR according to accepted selection criteria.

The rationale for our study was that some reports of very low uptake of PR in the post-hospitalization period may have reflected, in part, the fact that some COPD patients would not have been eligible for PR based on its standard selection criteria. For instance, in the Spitzer study cited earlier [5], 3321 of 223,832 patients (1.5%) initiated PR by 3 months. If our findings of 39% eligibility were applied to these statistics, the denominator would be reduced to 87,294 and the uptake at 3 months would increase to 3.8%. But even with this adjustment, PR uptake in the post-exacerbation period would still be extremely low, especially when taking into consideration its demonstrable benefits across multiple outcomes in this setting [11].

Individuals with COPD severe enough to warrant hospitalization often have prominent systemic effects from the disease such as frailty [12] and/or substantial comorbidity [13,14]. While systemic effects and coexisting conditions are targeted by and may improve with comprehensive PR, they may be severe enough to preclude the intervention—either by putting the individual at undue risk through participation or by substantially interfering with the rehabilitative process. Thus, systemic effects and comorbidity may be a double-edged sword: while they are responsible for much of the benefit from PR, they may be severe enough to serve as contraindications. The type and severity of these associated processes, while not necessarily precluding PR, may require a particular approach to it, such as center-based versus home-based with technology support.

Based on our review of discharged COPD patients’ records, only 39% were deemed eligible for PR. This number increased only slightly (45%) after eliminating from the analysis those with an incorrect diagnosis of COPD. This low eligibility for PR would decrease the denominator in uptake analyses and thereby mitigate to some extent the alarming findings in some studies, such as those mentioned above. However, even with this correction, the percentage of eligible patients participating in PR in the post-hospitalization period still remains very low, probably due to reasons outlined in the Introduction. Therefore, our study does not obviate the need to develop more ways to bring this valuable intervention to a wider audience.

We found two audits of PR eligibility for patients discharged after a COPD exacerbation. One study was performed in a single center in London and reported that 64% of patients met eligibility criteria for referral to PR [15]. The other was a retrospective analysis of data from two hospitals in Dublin that reported 54% eligibility after the elimination of those patients with incorrect diagnoses [16]. The reason(s) why our eligibility results were numerically lower are not clear, but may reflect analyses of different health care systems operating approximately a decade apart, or differences in demographics, types of comorbidities, or total disease burden. Of note, after elimination from analysis of those we deemed did not have COPD, our eligibility percentage increased slightly (44%), coming closer to the 54% cited above.

Our study had several limitations. While our analysis was based on data from two hospitals (one in an urban setting in a geographic area with low average income and the other in a suburban area with high average income), it remains unclear whether our PR eligibility results can be generalizable to other health care systems. Furthermore, our study design, which was based on a retrospective record review, did not allow for the determination of whether any of the 353 study patients actually participated in PR previously, during, or immediately after the hospitalization. However, we do not consider this a major limitation, as we focused only on potential eligibility, not uptake into a program.

Another limitation is that our eligibility and non-eligibility determinations for PR were based on review by investigators who were not directly participating in the patients’ care, relying only on clinical documentation that was present in the medical record at the time of the index hospitalization. In practice, this decision generally requires real-time decision-making and collaboration by health care providers, pulmonary rehabilitation coordinators, and other team members, and (in the United States) by the medical director of the program. Our retrospective review could not duplicate this complex process. However, we did employ a team of six experienced pulmonary clinicians, consisting of two pulmonologists and a nurse practitioner at each site. Notably, our full concordance in decision-making was high (73%), and the two independent teams identified remarkably similar eligibility determinations—38% and 40%.

Finally, the nature of our retrospective review allowed us to consider only criteria that would ordinarily preclude PR (i.e., exclusion criteria). The other component of the PR selection process—*does the individual have sufficient symptom burden, functional limitation, or health status limitation that would warrant PR?*—could not be directly assessed, as adequate documentation was often absent in the medical record. Eliminating those patients without sufficient severity for PR referral would only worsen our already low eligibility statistics. However, in general, if COPD is severe enough to require hospitalization, then PR may be considered an option, providing exclusion criteria are not present. Adding to this argument, the findings that PR given in the period following COPD hospitalization significantly reduces subsequent mortality and hospitalization risks in itself would likely be enough to recommend PR in this setting [2,17,18].

It is important to reiterate our study estimated only appropriate referrals to PR, not actual uptake into a PR program. The latter is often considerably lower than the former, as patients may opt out of treatment. For example, only about 75% of US veterans referred to PR started the intervention [19]. Reported barriers to uptake after referral, from a systematic review by Keating and colleagues [6], include interference or disruption of the patient’s usual routine, a negative influence by the referring physician, problems with program timing, access or transport issues, and an anticipated lack of benefit. In addition to deficiencies in the uptake of PR once appropriate referral is made, only approximately one-half to two-thirds of enrolled patients actually complete the program [20,21,22].

In summary, from our review of medical records of patients discharged from acute care hospitals with a principal discharge diagnosis of COPD exacerbation or respiratory failure secondary to COPD, only 39% met selection criteria for PR referral. We found a variety of factors contraindicating PR, including incorrect diagnosis of COPD; excessive severity of COPD and comorbid medical conditions; cognitive, psychiatric, or substance abuse issues; institutionalization post-discharge; and language barriers. While non-eligibility for PR in the post-hospitalization setting may mitigate to a small degree its strikingly low uptake percentage, PR remains grossly underutilized, and efforts to find ways to increase PR uptake—such as increasing referrals by clinicians or the institution of telehealth PR [23]—should continue.

## Figures and Tables

**Table 1:** Patient characteristics.

	Total	Hospital A	Hospital B
n	353	217	136
Age (years, mean ± SD)	71 ± 12	70 ± 11	72 ± 13
Male/Female (%)	47/53	52/48	46/54
Low SES (%)	40	54	20
Race: Black/White (%)	20/76	26/72	10/81

SES: socioeconomic status. General inclusion criteria for PR were persistent respiratory symptoms such as dyspnea or fatigue and/or functional status limitations despite otherwise optimal therapy [1].

**Table 2:** Non-eligibility categories.

Non-Eligibility Category	Total	Hospital A	Hospital B	*p*-Value
Incorrect diagnosis (%)	18	10	31	<0.0001
Severity of medical conditions (%)	44	40	50	0.06
Cognitive, psychiatric, or substance abuse issues (%)	24	27	19	0.10
Institutionalized post-discharge (%)	9	12	2	0.0007
Language barrier (%)	4	3	6	0.15

## Data Availability

The original contributions presented in this study are included in the article. Further inquiries can be directed to the corresponding author.

## Abbreviations

PR: pulmonary rehabilitation; COPD: chronic obstructive pulmonary disease; SAS: Statistical Analysis System.
